# Information compression exploits patterns of genome composition to discriminate populations and highlight regions of evolutionary interest

**DOI:** 10.1186/1471-2105-15-66

**Published:** 2014-03-07

**Authors:** Nicholas J Hudson, Laercio R Porto-Neto, James Kijas, Sean McWilliam, Ryan J Taft, Antonio Reverter

**Affiliations:** 1 Computational and Systems Biology, CSIRO Animal, Food and Health Sciences, St. Lucia, Brisbane, QLD 4067, Australia; 2 Institute for Molecular Bioscience, The University of Queensland, St. Lucia, Brisbane, QLD 4067, Australia

**Keywords:** Information compression, Phylogeography, Selection signatures

## Abstract

**Background:**

Genomic information allows population relatedness to be inferred and selected genes to be identified. Single nucleotide polymorphism microarray (SNP-chip) data, a proxy for genome composition, contains patterns in allele order and proportion. These patterns can be quantified by compression efficiency (CE). In principle, the composition of an entire genome can be represented by a CE number quantifying allele representation and order.

**Results:**

We applied a compression algorithm (DEFLATE) to genome-wide high-density SNP data from 4,155 human, 1,800 cattle, 1,222 sheep, 81 dogs and 49 mice samples. All human ethnic groups can be clustered by CE and the clusters recover phylogeography based on traditional fixation index (F_ST_) analyses. CE analysis of other mammals results in segregation by breed or species, and is sensitive to admixture and past effective population size. This clustering is a consequence of individual patterns such as runs of homozygosity. Intriguingly, a related approach can also be used to identify genomic loci that show population-specific CE segregation. A high resolution CE ‘sliding window’ scan across the human genome, organised at the population level, revealed genes known to be under evolutionary pressure. These include *SLC24A5* (European and Gujarati Indian skin pigmentation), *HERC2* (European eye color), *LCT* (European and Maasai milk digestion) and *EDAR* (Asian hair thickness). We also identified a set of previously unidentified loci with high population-specific CE scores including the chromatin remodeler *SCMH1* in Africans and *EDA2R* in Asians. Closer inspection reveals that these prioritised genomic regions do not correspond to simple runs of homozygosity but rather compositionally complex regions that are shared by many individuals of a given population. Unlike F_ST_, CE analyses do not require *ab initio* population comparisons and are amenable to the hemizygous X chromosome.

**Conclusions:**

We conclude with a discussion of the implications of CE for a complex systems science view of genome evolution. CE allows one to clearly visualise the evolution of individual genomes and populations through a formal, mathematically-rigorous information space. Overall, CE makes a set of biological predictions, some of which are unique and await functional validation.

## Background

The history of life is written in genomes. Evolutionary forces and historical artefacts leave discernible footprints on DNA sequence, and their identification and subsequent interpretation is an active area of genetic research. Single nucleotide polymorphism microarray (SNP-chip) data capture within species variation. SNP data are typically used to investigate the genetic origin of diseases and other phenotypes, identify genetic differences between populations, and to infer the shared evolutionary history of those populations [1]. These inferences are usually derived from analyses of percent heterozygosity (HET), explorations of allele frequency using fixation index (F_ST_) and principal component analysis (PCA) [1]. While being one of the most recognized and commonly implemented metrics, the use of F_ST_ to quantify the genetic distance between populations is not free of challenges. It has been demonstrated [2] that the choice of estimator, the method of combining estimates across SNPs, and the scheme of SNP ascertainment can impact the F_ST_ results. Similarly, according to [3] a weakness of F_ST_ is that it implicitly assumes that populations have the same effective size and were independently derived from the same ancestral population. If this assumption is violated, which is often the case, genome scans can yield false positives. Strengths and weaknesses of applying PCA to population genetics are discussed in detail elsewhere [4,5].

The linear data strings produced by SNP-chips possess two very simple numerical properties – the order and proportion of 0’s, 1’s and 2’s (corresponding to the three possible genotypes for a given bi-allelic SNP). These compositional numerical properties have not been fully explored but may be amenable to novel pattern recognition analyses thereby yielding unique new biological insights. It has long been recognized that regularities in data structure enforce statistical redundancies that can be quantified by the related concepts of entropy and compression [6]. This compressibility is a proxy for the minimum amount of information required to reproduce a data set, i.e. its Kolmogorov complexity [7]. The compression output can be queried to identify patterns that may have otherwise remained obscure. Clustering large data sets by compression metrics has been successfully applied to whole mitochondrial genome phylogenetic reconstructions [8], microbiota composition comparison [9], interpretation and segregation of gene expression data [10], virus phylogeny, relatedness of languages, and even clustering of musical genres [11].

Here we use dense genome-wide SNP-chip data as a proxy for genome composition, which facilitates exploitation of broad patterns of genomic organization relating to the order and proportion of homozygote and heterozygote genotypes. By expressing the compressed file size to its uncompressed form one gets a measure of Compression Efficiency (CE). This output reflects data regularities in a convenient manner. Our approach spans datasets derived from five mammalian species, and includes humans and several domestic animals. The inclusion of domestic animals allowed us to explore genomic patterns in populations with well-defined phenotypes, varying periods and intensities of artificial selection, and variations in past effective population size [12]. We show that CE analyses can be successfully deployed to discriminate between populations and breeds in a computationally efficient manner that has an appealingly simple methodology.

## Results

### Genome-wide compression efficiency reveals population structure

In this study SNP data were used as a proxy for genomic DNA sequence composition. The data regularities exploited by the compression efficiency metric were derived from the patterns of allele order and proportion across both individuals and populations. Our expectation is that SNP data are dense enough to provide a reasonable reflection of overall genomic composition. However, some clear differences between real sequence versus SNP data include the absence of DNA repeat regions on SNP arrays. Furthermore, SNP are intended to capture allele variation relative to a reference, and in this sense are abstracted from raw genome sequence. Finally, caution is needed as SNP ascertainment bias may complicate interpretation across populations.

**Figure 1:**
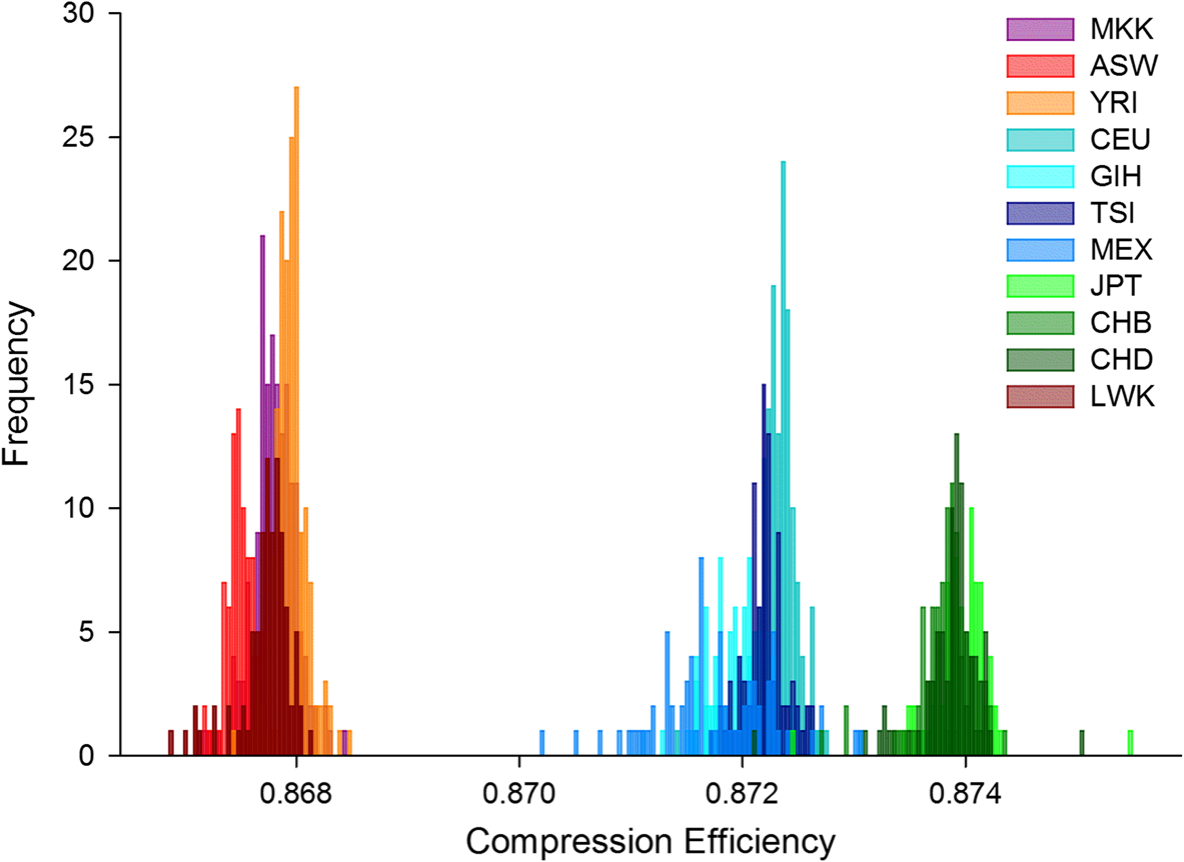
**Compression efficiency discriminates human populations.** Empirical density function of compression efficiency calculated using *gzip*. The eleven human populations from HapMap 3 are discriminated. The clusters resonate with known phylogeographic relationships. The Han Chinese in Beijing (CHB), the Chinese in metropolitan Denver (CHD) and the Japanese in Tokyo (JPT) are co-located by high compression efficiency, and conversely the South-West USA with African ancestry (ASW), the Luhya in Kenya (LWK), the Maasai in Kenya (MKK) and the Yoruba in Nigeria (YRI) are co-located by low compression efficiency. The middle cluster encompasses the Mexican (MEX), Indians (GIH), Italian (TSI) and central Europeans (CEU).

**Figure 2:**
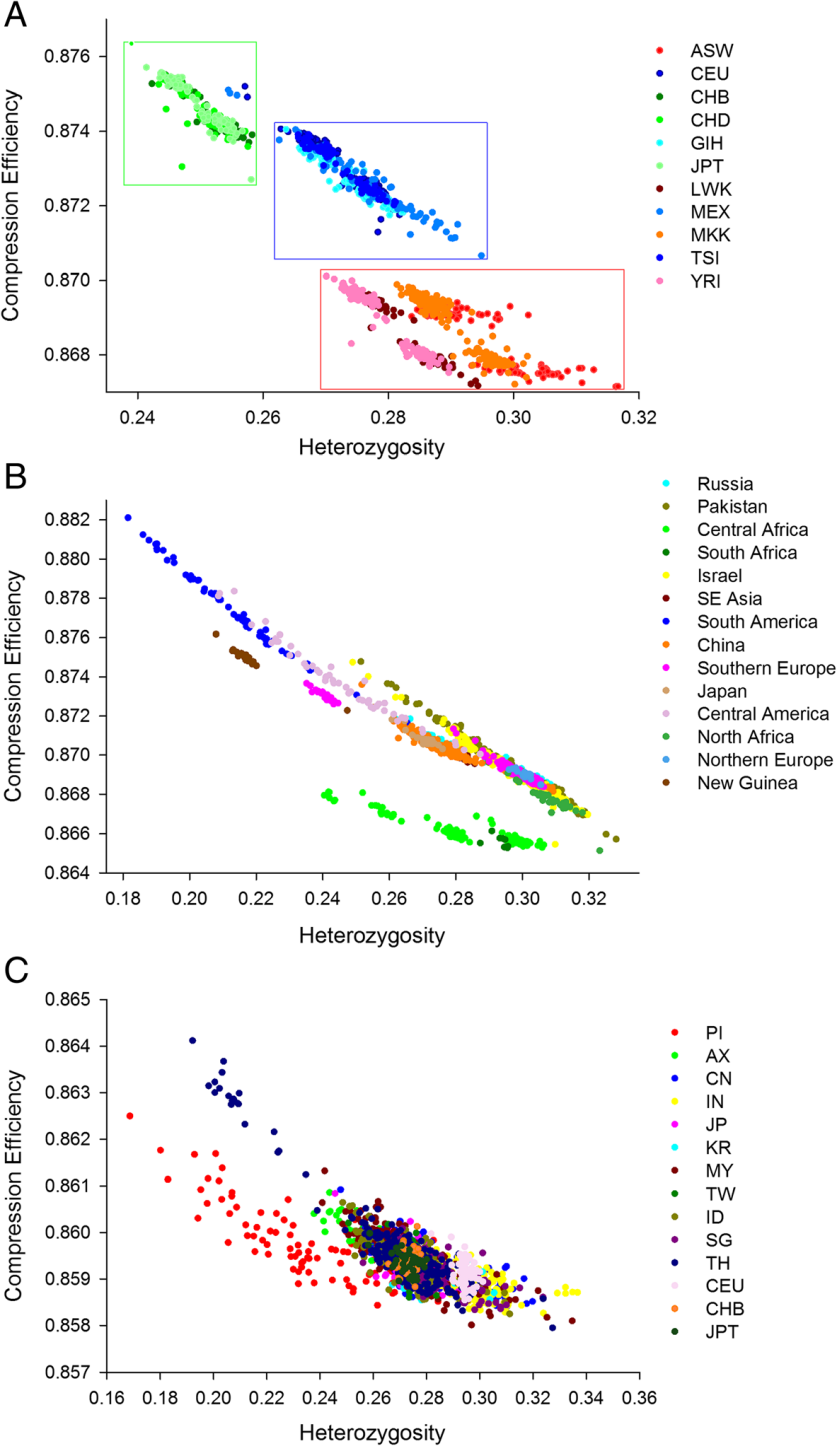
**Compression efficiency against heterozygosity resolves human populations.** Compression efficiency (y-axis) against heterozygosity (x-axis) for the three human data sets: **(A)** HapMap 3 (1,184 individuals across 11 populations), **(B)** HGDP (1,043 individuals representing 51 populations from 14 geographical regions) and **(C)** Pan-Asia (1,928 individuals across 75 populations). We find that plotting the genome-wide compression efficiency data versus genome-wide heterozygosity reinforces the strength of the population discrimination in all cases. Although the broad relationship is negative, some populations clearly have similar genome-wide heterozygosity but very different compression efficiency. The spatial resolution is consistent with previous phylogeographic reconstructions based on Fixation Index and Principal Components Analysis, illustrated by the coloured boxes in the first panel representing Asian and Africans as extreme with the Europeans intermediate.

The CE output for some exemplar data strings are shown in Table 1. They reveal that the two properties driving data regularity (and therefore CE) - order and proportion - bear a strong mutual relationship with each other. High levels of proportional bias will serve to enforce bias in order, and vice versa. In contrast, Shannon’s entropy [6], an information theory statistic for measuring regularities in data streams, exploits proportion. Like Shannon’s entropy, CE captures proportion, but it also reflects sequence regularity in scenarios where Shannon’s entropy is uninformative (Table 1), because Shannon’s entropy does not exploit patterns relating to order.When genome-wide CE values were obtained for each individual from the 11 human populations from HapMap3, it successfully sorted individuals into their population of origin with varying degrees of overlap (Figure 1, Figure 2A). Although the spread of the data is narrow, it clearly segregates the populations such that the African show low CE values and high heterozygosity, while the Asian populations have high CE values and low heterozygosity. Indeed, all populations within the HGDP and Pan Asian data were discriminated to some extent by the CE approach, yielding population resolution in all cases (Figure 2B, C). Comparing all human SNP data sets, the indigenous Brazilian populations (i.e. the Surui and Karitiana) exhibited the highest CE.

**Table 1:** **The rationale behind Compression Efficiency** (**CE**)

**Sequence based on 30 SNPs**	**Rationale**	**CE,%**
000000000011111111112222222222	10 “0” + 10 “1” + 10 “2”	38.33
012012012012012012012012012012	10 “012”	40.00
001202020022111221100211121200	Random location of 10 “0”, “1” and “2”	11.66
000000000011111111112222222222	10 “0” + 10 “1” + 10 “2” replicated 5 times	75.48
000000000011111111112222222222		
000000000011111111112222222222		
000000000011111111112222222222		
000000000011111111112222222222		
012012012012012012012012012012	10 “012” replicated 5 times	76.13
012012012012012012012012012012		
012012012012012012012012012012		
012012012012012012012012012012		
012012012012012012012012012012		
001202020022111221100211121200	Random location of 10 “0”, “1” and “2” replicated 5 times	67.10
001202020022111221100211121200		
001202020022111221100211121200		
001202020022111221100211121200		
001202020022111221100211121200		
001202020022111221100211121200	5 different random locations of 10 “0”, “1” and “2”	40.64
112220101200102022010110102212		
210200221211112120020122001010		
120221110000202110122021012210		
210010201112220012100101222012		

Compression efficiency for hypothetical sequences based on 30 SNPs for one individual (first three rows) or five individuals (last four rows). In all cases the Shannon’s Entropy would be identical i.e. 1.585 (= -3 (1/3) log_2_(1/3) ) because each SNP call is equi-probable. Compression efficiency exploits patterns in order as well as proportion allowing it to discriminate data that cannot be discriminated by Shannon’s entropy. The Rationale column is a verbal approximation of the algorithmic complexity. Regular sequences have a small algorithmic complexity and high Compression efficiency. However, complex, irregular sequences embedded in a homogeneous population can still exhibit a relatively high Compression efficiency %. Our sliding window heterozygosity corrected compression efficiency approach exploits this combination (the last four rows). In effect, a population-level assessment is made of a region’s sequence entropy, detecting high co-sharing of even very complex regions.

### Relationship between CE and F_ST_

**Figure 3:**
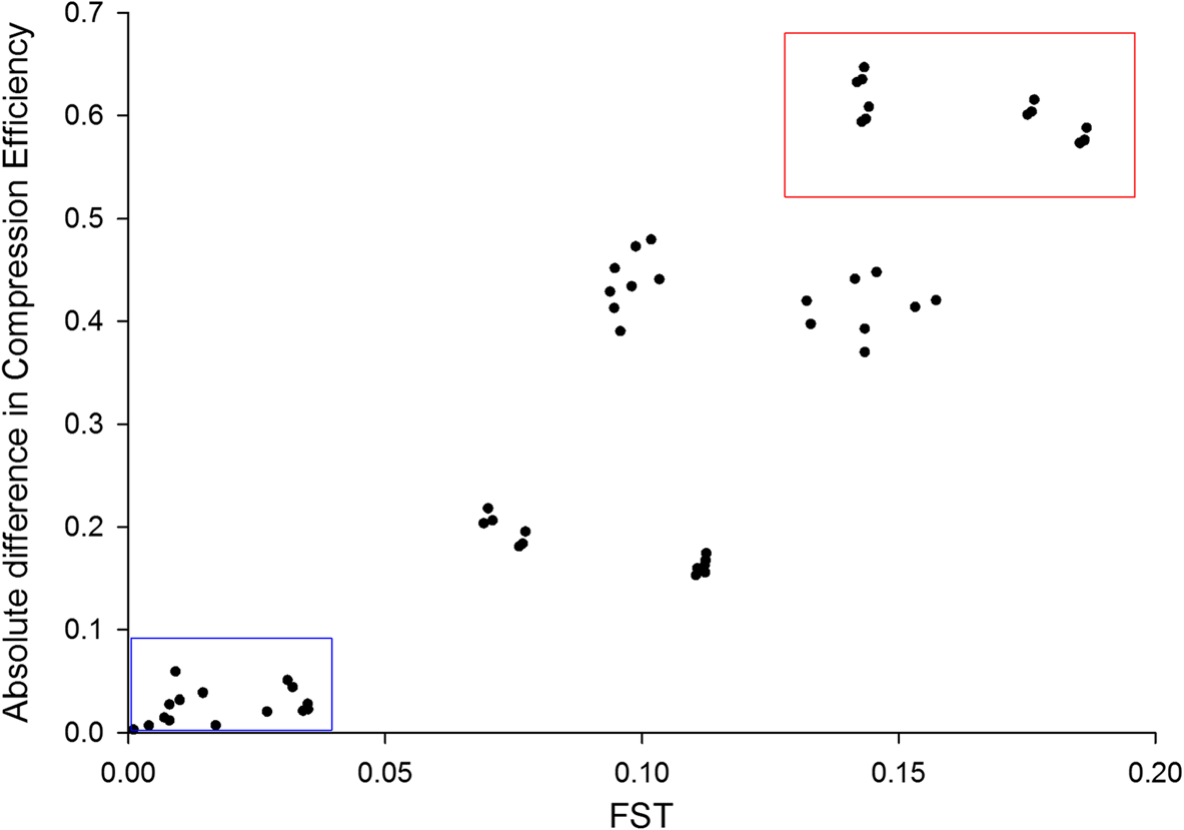
**The positive correlation between absolute difference in CE and F**_**ST **_**suggests CE accurately reflects aspects of population structure.** Each of the 55 dots represents every population pair from the 11 human Hapmap populations. The blue box encapsulates closely related populations i.e. comparisons between two Asian, two African or two European populations. The red box encapsulates the most distantly related populations i.e. the comparisons between the various Asian and African populations.

The correlation between F_ST_ and CE is 0.885 implying CE reflects to a large extent the population resolution of F_ST_. The pairwise relationship for all 55 population pairs can be found in Figure 3.

Given that only a fraction of the human genome is protein-coding, and that canonical genes are on average more highly conserved and have a substantially lower mutation rate [13], we explored the possibility in the human HapMap data that human protein-coding and non-protein-coding regions would segregate by CE. We would normally expect an increase in CE to correspond with a decrease in heterozygosity. For all populations, coding regions have lower HET than non coding regions (Additional file 1: Figure S1A). However, when examining CE of the low HET coding regions in isolation the expected increase in CE was only observed in the African populations (Additional file 1: Figure S1B). For the remainder, i.e. all the non-African populations, there was no increase in CE when examining coding regions in isolation.

We find that in all human populations there is a relationship between high heterozygosity and low CE. This is because a more even proportion of 0’s, 1’s and 2’s (approaching 33% heterozygosity) naturally increases the entropy of the data stream, for the same reason the outcome of a 3-sided dice roll is less certain than that of a 2-sided dice roll. However, heterozygosity does not fully account for the CE output because 1) much genomic regularity is a product of order in combination with proportion and 2) the percentage of 1’s does not exclusively govern either the order or proportion of 0’s and 2’s that comprise the remainder. We also note that in addition to being easier to implement than F_ST_ the CE approach was computationally more efficient, taking 16 seconds compared to 94 seconds.

**Figure 4:**
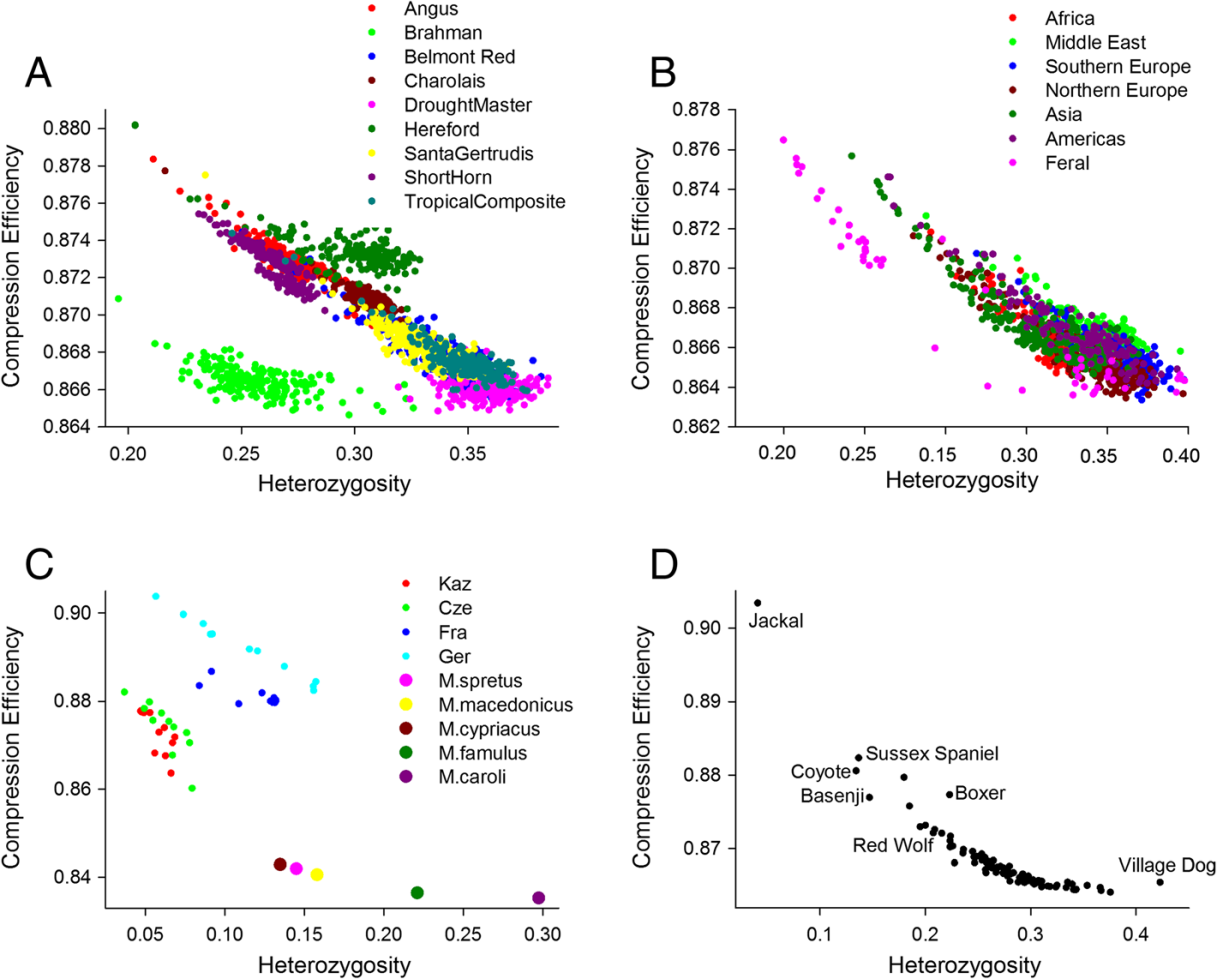
**Compression efficiency against heterozygosity resolves non-human populations.** Compression efficiency (y-axis) against heterozygosity (x-axis) for the four non-human data sets: **(A)** cattle (n = 1,800), **(B)** sheep (n = 1,222), **(C)** mouse (n = 49) and **(D)** dog (n = 83). The discrimination afforded by genome-wide compression efficiency and heterozygosity is effective in all species under consideration. Inclusion of wild as well as domestic populations shows the compression efficiency is sensitive to selection pressures, past effective population sizes, pedigree structures, different heterozygosities and different levels of linkage disequilibrium. The Brahman are the only Indicine cattle in panel A, the remainder being Taurine breeds.

Analysis of non-human populations demonstrated that CE was able to successfully reveal known aspects of population history (Figure 4). Large SNP datasets were available from cattle and sheep populations, where multiple animals per breed were drawn from a diversity of breeds and geographic regions. Clear evidence for population substructure was evident in cattle, which is composed of two sub-species (*Bos taurus* and *Bos indicus*) (Figure 4A). CE distinguished Brahman cattle (*Bos indicus*) from all other populations. The combination of CE and heterozygosity separated populations on the basis of their ancestral genomic composition. For example, breeds with a high proportion of *Bos indicus* ancestry (Droughtmaster and Santa Gertrudis) had high heterozygosity and low CE, while purebred taurine breeds (Charolais, Angus and Shorthorn) showed generally higher CE and reduced heterozygosity.

Domestic sheep are not composed of separate sub-species and have a population history characterized by frequent gene flow between breeds [14]. This generally weak population substructure (compared with cattle and dogs) was reflected in the data as domestic breeds from six major geographic regions were positioned in a single large undifferentiated cluster (Figure 4B). Feral sheep, however, clustered separately from modern domestic breeds, due to their low heterozygosity and high CE. The mouse population data shows variation in the CE and heterozygosity relationship (Figure 4C). The German mouse population is arranged in a linear fashion whereas other populations are arranged more diffusely. This implies that at a whole genome level the German mice possess a particularly tight relationship between CE and heterozygosity, although the heterozygosity itself is variable. Like the mice data, the dog data is based on a single representative individual for each breed (Figure 4D). The extreme position of the village dog (rural, free-ranging), considered to be neither wild nor feral, is noteworthy. Taken together, CE and heterozygosity recapitulated known aspects of population history for each animal species tested.

### High resolution sliding window CE reveals regions that discriminate populations including signatures of selection

Given the ability of genome-wide CE to recreate phylogeographic relationships, we investigated if CE could be employed to identify individual genomic loci that showed significant deviations in allele order and composition. We speculated that since positive selection is known to leave detectable signatures in genomic patterns of variability [15], CE may be able to not only identify regions under common constraint across human populations, but also loci that differed systematically between populations. In this analysis a heterozygosity corrected CE (CEh) score, which was subsequently normalized (to allow for cross-population comparisons) by its Z-score (i.e. CEhZ), was computed for sliding windows of 50 SNPs across all 11 human HapMap populations. Regions of interest were identified by taking SNPs with CEhZ scores 3-fold higher than the mean, and clustering SNP within 20 Kb of one another.

We identified ~450 regions per population with statistically significant CEhZ scores, with an average size ~97 kb (hereafter “CEhZ loci”). Analysis of CEhZ loci revealed that their distribution varies between populations (Additional file 2: Figure S2A) and that most of them have substantial heterozygosity and so do not correspond to simple runs of homozygosity (Additional file 2: Figure S2B). Rather, the CEhZ loci include complex mixes of homozygotes and heterozygotes that are co-shared by many members of a population.

Intriguingly, only nine CEhZ loci were common across all 11 human HapMap populations. These contained retinoblastoma 1 (*RB1*, tumour suppressor and cell-cycle regulator), dihydropyrimidine dehydrogenase (*DPYD*, pyrimidine catabolic enzyme), histone deacetyase 1 (*HDAC1*, chromatin remodeller) citrate synthase (CS, pace-making enzyme in the first step of the Citric Acid Cycle), peroxisome proliferator-activated receptor delta (*PPARD*, nuclear hormone receptor that controls the size and number of fat metabolizing peroxisomes and mitochondria), and Neutral alpha-glucosidase C (*GANC*, a key enzyme in glycogen metabolism) among others (see Additional file 3: Data S1) – all genes involved in fundamental cellular, developmental and metabolic processes. Since the CEhZ metric is detecting population-level regions of homogeneity, it is tempting to speculate that CEhZ loci common to all human populations are coincident with regions of fundamental importance to the human lineage.

**Figure 5:**
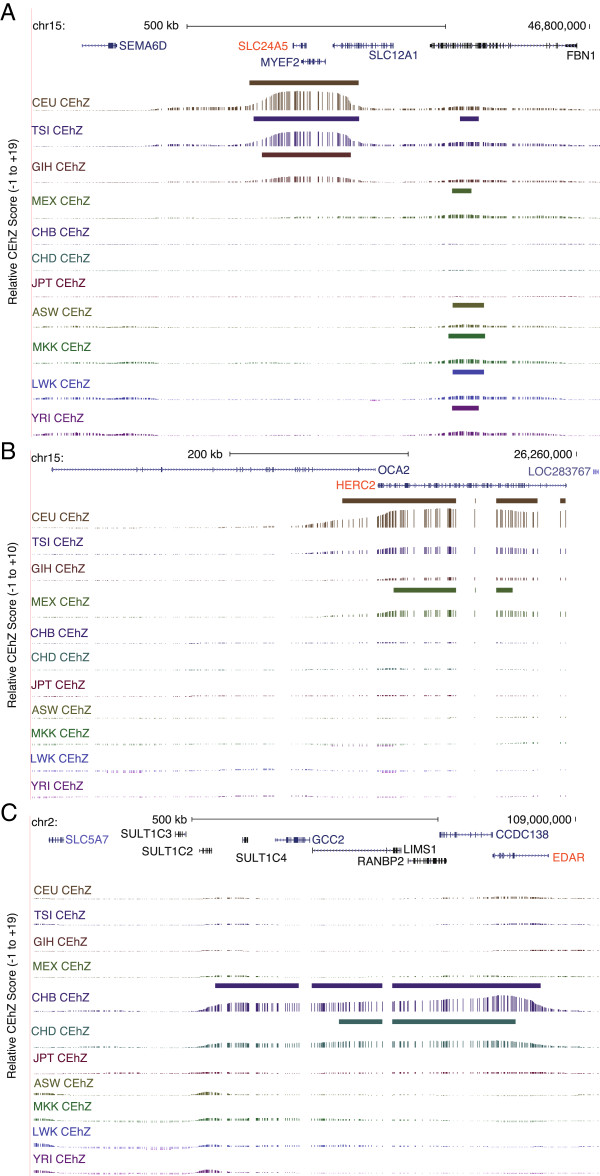
**CEhZ on an overlapping sliding window basis clearly highlights known signatures of selection in humans. (A)** Skin lightening in Europeans (*SLC24A5*), **(B)** Blue eye colour in Europeans (*HERC2*) and **(C)** Hair texture in Asians (*EDAR*).

To investigate if high CEhZ scores may be indicative of evolutionary pressure, CEhZ loci were intersected with regions recently identified as harbouring signals of natural selection in central Europeans (CEU), Chinese (CHB) or Yoruba Africans (YRI) using the composite of multiple signals (CMS) test [16]. This revealed that 20% of the CEU, 13% of the YRI, and 10% of the CHB regions identified by CEhZ and CMS overlapped (Additional file 4: Data S2). Importantly, this set contains loci that are exemplars of natural selection in modern humans. For example *SLC24A5* which is associated with skin lightening in cold climate Europeans [17] Tuscans and Gujarati Indians [18] is specifically detected by CEhZ in the CEU, TSI and GIH populations (Figure 5A); *HERC2 is* associated with variation in eye colour in Europeans [19] and is similarly detected by CEhZ specifically in CEU and MEX (Figure 5B); and *EDAR* has recently been associated with variation in hair texture in Asians [20], and is detected in both Chinese populations but not Japanese (Figure 5C).

**Figure 6:**
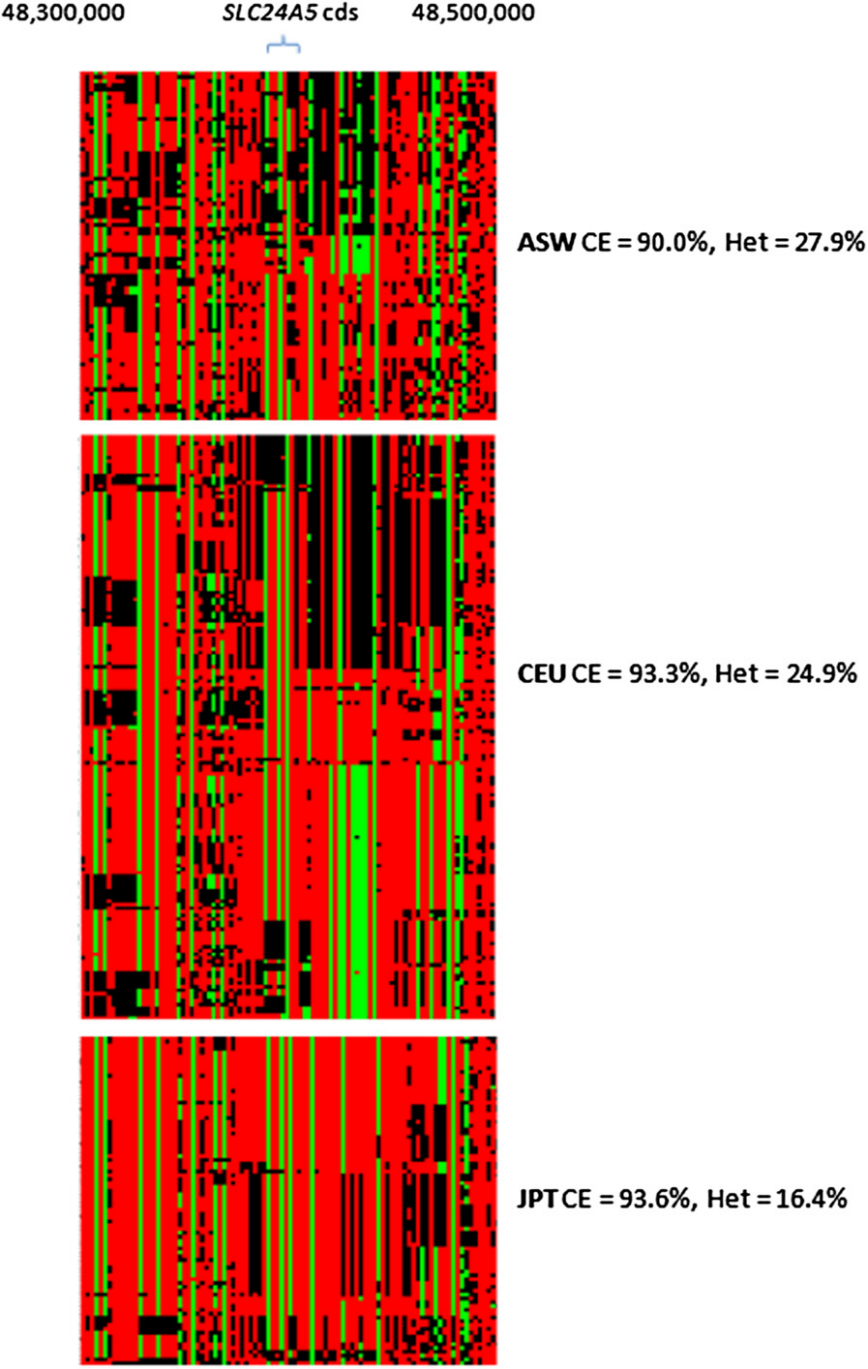
**The detailed allele composition of the *SLC24A5 *skin pigmentation region in dark skinned ASW versus lighter skinned CEU and JPT.** The alleles are represented by color as follows: red = 0, green = 2, black = 1. Population-level regularities in allele order are very evident in both European and Japanese populations but not South West with African Ancestry. The Japanese were included for illustration purposes. Their compression efficiency was no higher than the CEU despite a much lower heterozygosity. Following correction for heterozygosity no peak was detected over this region. The region exactly overlying the *SLC24A5* coding sequence (Chr15:48,413,169 – 48,434,589) is coincident with almost identical allele patterns in both European and Japanese. Downstream of the coding sequence both European and Japanese possess population-level regularities in composition, but the regularities differ.

A detailed example of the sort of population-level shared patterns identified by CEhZ is illustrated for the skin lightening *SLC24A5* region in light skinned CEU compared to darker skinned ASW (Figure 6). We created three heatmap matrices, clustered on rows. Patterns of population sub-structure based on haplotypes are clearly evident within the CEU and JPT, as are systematic differences between the three populations. The darker skinned ASW are much more heterogeneous (entropic) in this region at the population-level than the other two populations. A substantial reduction in HET is apparent in the JPT but not the CEU. It is noteworthy that the CEU show a compression peak as strong as the JPT, but without the reduction in HET.

**Figure 7:**
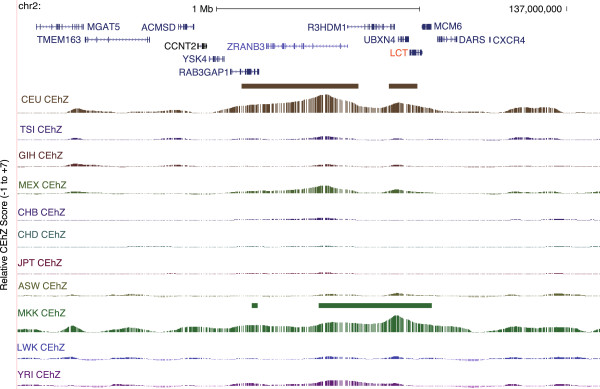
**CEhZ on an overlapping sliding window basis clearly highlights the signature of selection over lactase.** Adult lactase persistence following co-evolution with livestock in Europeans and pastoral Maasai Africans (*LCT*).

Intriguingly, CEhZ analysis reveals peaks for both the CEU and the Maasai overlapping *LCT*, or lactase (Figure 7). We note that the CEhZ data show a more pronounced peak in the Maasai compared to CEU.

**Figure 8:**
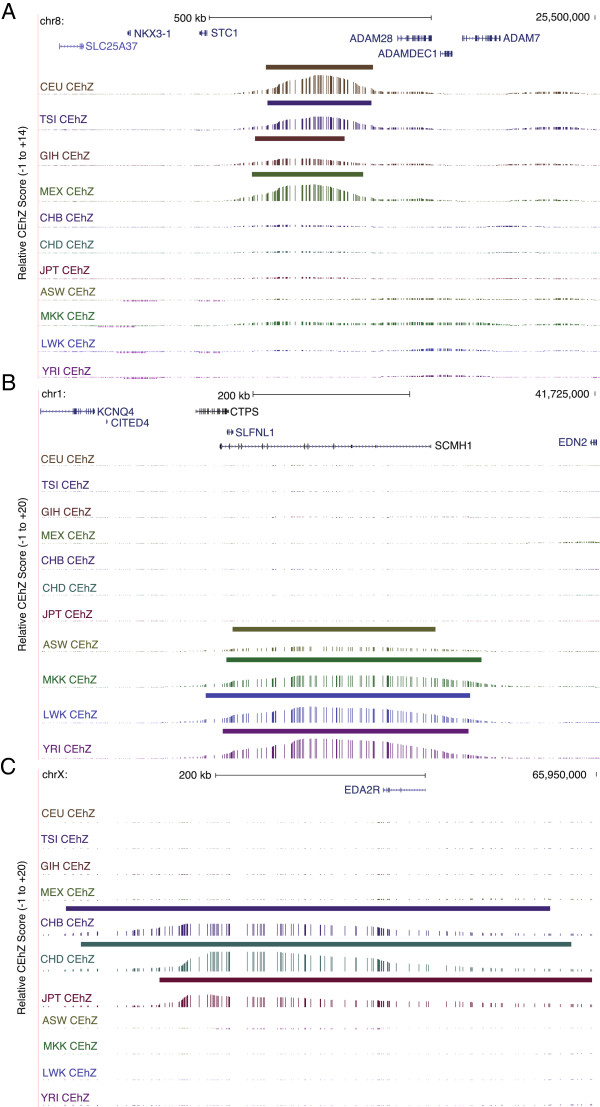
**CEhZ on an overlapping sliding window basis makes unique new predictions of regions of evolutionary interest in humans. (A)** A European and Mexican peak in a gene desert containing a long non-coding RNA transcribed preferentially in placenta **(B)** An African peak over a gene encoding a chromatin remodeller (*SCMH1*) and **(C)** An Asian peak over *EDA2R* encoded on the X chromosome.

To identify high-confidence CEhZ loci that may warrant further investigation, regions specific to population clusters were collated. This revealed 162 African (ASW, MKK, LWK, YRI), 47 European (CEU, TSI and GIH), and 69 Asian (JPT, CHB, and CHD) CEhZ loci. These sites include a European CEhZ peak that corresponds to an apparent “gene desert” containing a long non-coding RNA of unknown annotated function (Figure 8A) preferentially expressed in placenta (*data not shown*), an African peak (Figure 8B) over a chromatin remodeller and polycomb group protein that has been previously associated with genetic determinants of height (*SCMH1*) [21], and an Asian peak (Figure 8C) over the X chromosome gene, *EDA2R*, which encodes the ectodysplasin A2 receptor and neighbours the androgen receptor. These regions have been uniquely identified by the CEhZ approach. We hypothesize that further investigation of these loci will reveal a possible role in population-specific biological processes.

**Figure 9:**
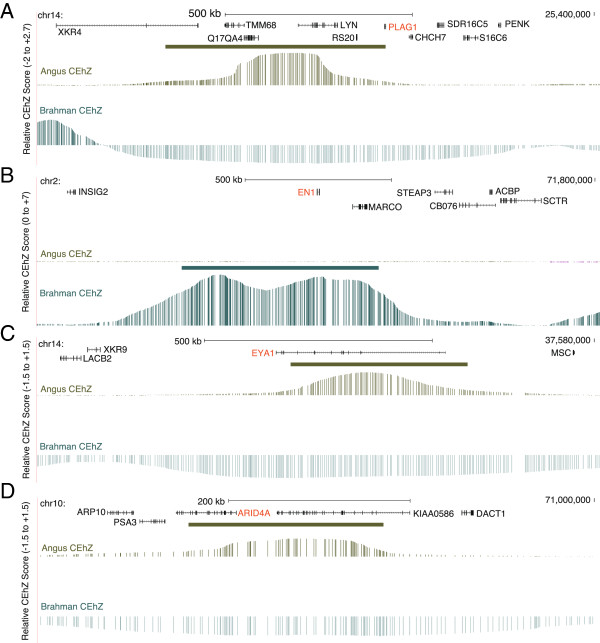
**CEhZ applied to Brahman and Angus cattle. (A)** Contrasting the heterozygosity corrected compression efficiency of Brahman and Angus coincides with a known signature of selection around the 25 Mb region of bovine chromosome 14 incorporating the gene *PLAG1* associated with tropical adaptation and a number of phenotypic traits such as fertility and growth. New regions predicted include *EN1* for Brahman **(B)**, *EYA1* for Angus **(C)** and *ARID4A* for Angus **(D)**.

Extending the CEhZ approach to two cattle species, Angus and Brahman, comparing to the literature revealed that 16 of 30 the most extreme CEhZ loci overlap with genomic regions previously described as being under selection. Similarly, in an analysis equivalent to the human sliding window approach we found 6 of the top 10 regions also have been previously documented as harbouring signatures of selection (see Table 2) [22-27]. Furthermore, we also identified CEhZ loci overlapping with known QTL for production traits, including BTA14:~25 Mb which contains the *PLAG1* gene [28-30] (Figure 9A) previously associated with cattle growth and fertility [28-30]. We have highlighted 3 other regions that also discriminate the breeds, in each case coincident with genes encoding proteins of fundamental developmental importance: *EN1* (Figure 9B) plays a role in central nervous system pattern formation [31], *EYA1* (Figure 9C) plays a role in muscle fibre composition [32] and *ARID4A* (Figure 9D) plays a role in male fertility [33].

**Table 2:** **Compression efficiency identifies bovine signatures of selection**^1^

**Chr**	**Mbp**	**Angus**			**Brahman**			**Genes (300 Kb up and down)**	**Cross ref**
		**Het (%)**	**CE**	**Z**-**score**	**Het (%)**	**CE**	**Z**-**score**		
2	62.1	8.596	0.976	2.886	22.053	0.962	0.622	*DARS*, *LCT*, *MCM6*, *R3HDM1*, *UBXN4*, *ZRANB3*	[22,34,35]
3	28.5	1.499	0.985	57.316	33.883	0.949	-0.798	*AMPD1*, *CSDE1*, *NGF*, *NRAS*, *SIKE1*, *TSPAN2*	-
5	47.9	30.669	0.961	-0.014	5.737	0.977	13.380	*GRIP1*, *HELB*, *IRAK3*, *LLPH*, *TMBIM4*	[22,23,34,36,37]
5	121.1	31.960	0.936	-0.023	9.453	0.956	6.100	*ALG12*, *CRELD2*, *MLC1*, *PANX2*, *TRABD*	*
6	77.6	9.009	0.976	0.259	13.584	0.963	4.293	*LPHN3*	-
12	24.7	26.202	0.953	-0.004	12.780	0.958	4.202	*ALG5*, *EXOSC8*, *FAM48A*, *POSTN*, *RFXAP*, *SMAD9*	[34]
19	63.9	27.905	0.945	-0.013	8.545	0.965	7.798	*CACNG1*, *CACNG4*, *CACNG5*, *HELZ*	[34]
22	51.3	33.490	0.936	-0.026	9.342	0.963	6.091	*AMT*, *APEH*, *ARIH2*, *CCDC36*, *CCDC71*, *DAG1*, *IMPDH2*, *IP6K1*, *KLHDC8B*, *LAMB2*, *MST1*, *NICN1*, *P4HTM*, *QARS*, *QRICH1*, *RHOA*, *RNF123*, *TCTA*, *USP19*, *USP4*, *WDR6*	[24,34]
X	39.1	0.644	0.982	1.185	3.639	0.964	1.536	*DKC1*, *F8*, *FUNDC2*, *GAB3*, *KIR3DL2*, *MPP1*, *MTCP1*	*
X	86.9	0.081	0.986	3.149	5.991	0.951	-0.111	*EFNB1*, *PJA1*, *STARD8*, *YIPF6*	[24]

^1^Heterozygosis, Compression Efficiency and Z-scores values are averages of a window (+/- 300 Kb) from the reference position.^*^The SNPs giving the signal in these regions were unassigned on the bovine assembly Btau4.2.

Enomic regions with noteworthy (Top 1%) Z-score normalized heterozygosity corrected compression efficiency across bovine Brahman and Angus populations.

Unlike F_ST_ CEhZ is computed on a within genome basis. No contrasting population is required (although clearly it is informative to contrast CEhZ loci across populations), which means the analysis can be run on newly genotyped populations in isolation, identifying regions of interest even in species whose genome has poor functional annotation. Furthermore, CEhZ can also be computed on the mammalian X chromosome whose hemizygosity in males normally precludes population-level analyses by conventional methods.

The CEhZ human and bovine data (both SNP-level scores and CEhZ loci) are available as a hub that can be visualized using the UCSC Genome Browser: https://surf.genome.at.uq.edu.au/~uqrtaft/CEhZ/hub.txt).

### Defining the compression efficiency parameter space

In the discipline of complex systems science there exists the concept of ‘The Edge of Chaos’ capturing the properties of the phase transition that exists between order and chaos. This transition zone is important because it has been found to possess desirable evolutionary and computational properties.

**Figure 10:**
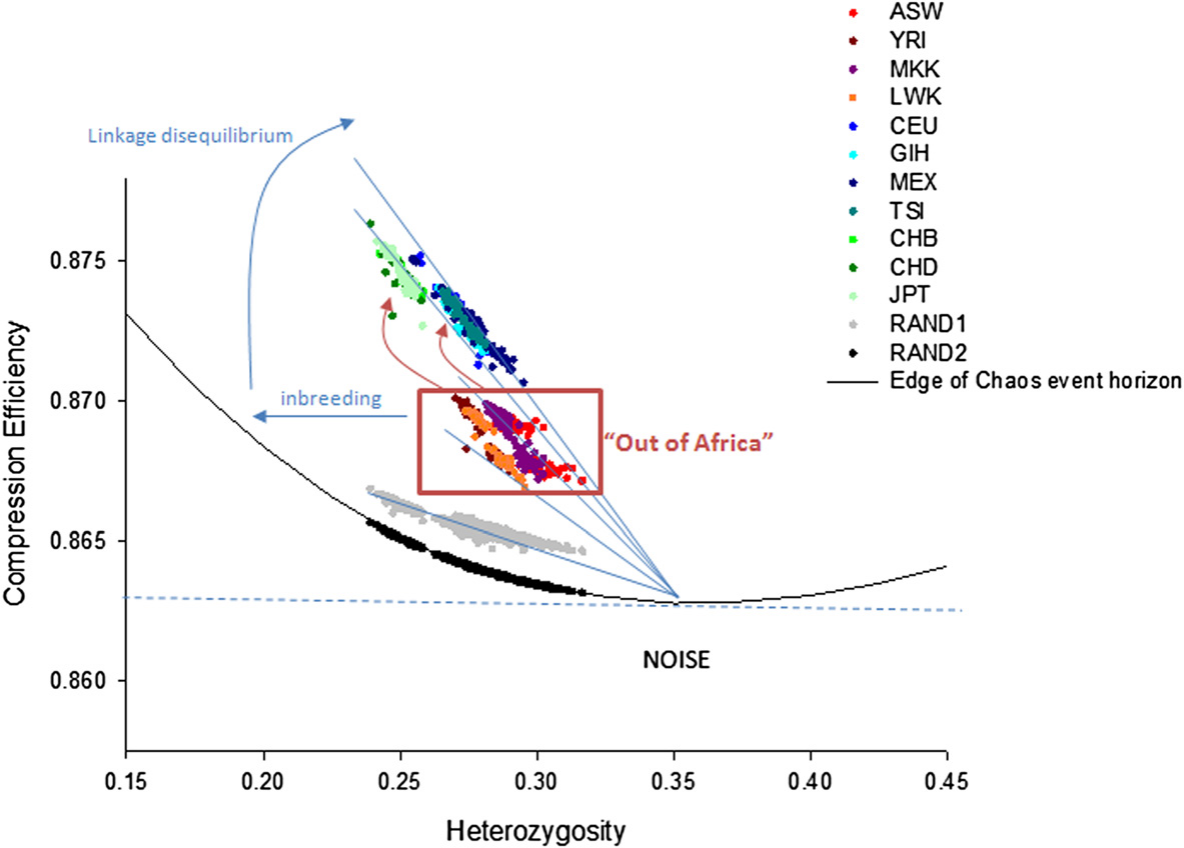
**Visualising genomes evolution in information space.** The curved black line represents the line of noise as defined by RAND2. The more regular the genomic data, the greater the distance above the black line. All real genomes are just above the black line, indicating their proximity to the phase transition between order and chaos. Furthermore, the populations are aligned to the lowest point of the curve. The relatively basal position of the African populations (surrounded by the red box) in this directional scheme appears consistent with the “Out of Africa” hypothesis.

It did not escape our notice that the CE approach allows one to formalise and test this concept in the context of genomics. One can scramble the data, measure the CE consequences, and compare it to the informational properties of the real genomes. We scrambled the order of SNPs using both the proportions from that particular individual (RAND1) but also a deeper randomization based on proportions derived from the population at large (RAND2). RAND2 is valuable in defining the parameter space we are interested in, as it can be explored for the entire range of conceivable heterozygosity i.e. from 0 to 100. Here, 100% produces a highly compressible string of 1’s while 0% is composed of a random string of 0’s and 2’s only. Because this RAND2 sequence is maximally chaotic the resultant curve defines the signal to noise threshold for every level of heterozygosity. Intriguingly, it can be observed that the real mammalian DNA sequences align to, or emanate from, the point of minimum CE coincident with ~33% heterozygosity (Figure 10; Additional file 5: Figure S3).

The outcome is similar across species. That is, the CE of genomes always aligns with the point of minimum CE. Superimposed in Figure 10 are lines intended to elucidate possible mechanisms for driving genomic movement through information space. Overall, this creates an impression that genomes evolve from (or to) the point of minimum CE, reflecting the combined impacts of inbreeding, LD (linkage disequilibrium) and other unidentified factors. There is a strong negative relationship between CE and HET. However, through outbreeding, HET values beyond 33% can be achieved and, in this region increases in HET can be associated with increases in CE. From a numerical perspective, *proportion*-based regularity (bias) increases as one moves farther away from the ~33% HET (the point of equilibrium) on both sides of the x axis, while *order*-based regularity (pattern) increases as you increase CE moving upwards on the y axis.

## Discussion

Given that the purpose of genomes is to encode and transact information, it is not surprising that principles from Information Theory have been previously used to quantify their informational properties [38-46]. However, the historical use of compression *per se* in genomics has been more from a practical and technical perspective [47,48], such as how to store large datasets in an efficient manner, rather than that of pattern recognition and biological interpretation. The only previous example of clustering by compression in the genomic area that we are aware of is the mitochondrial work of [8] which reconstructed eutherian and mammalian phylogenies.

Here, we have illustrated that CE analysis of SNP data allows one to 1) discriminate populations reconstituting known aspects of their diversity, 2) identify at very high resolution genomic regions of evolutionary interest including validated signatures of selection 3) visualise evolution through informational space and 4) achieve these goals using a method that has an appealing logical simplicity. The hypothesis-free pattern recognition qualities of CE allow exploitation of *order* as well as *proportion*, the two sources of regularity in any data stream. This is important because the detection of patterns can be used to cluster data based on guilt-by-association and drive the inference of biological meaning.

One conceptual advantage of using a proxy for direct genetic evidence (CE) rather than the direct evidence itself (such as F_ST_) is that previously unrecognized informational footprints may be present in the data which we may wish to exploit but whose expected properties we do not need to identify, nor fully understand, *a priori*.

Overall, we document a promising new perspective on analysing genomic data which is intended to be complementary to existing mathematical approaches, not to supplant them. Given this is the first publication of a wholly new approach, we are not yet in a position to formally connect our ideas to existing population genetics theory in a rigorous mathematical sense. Nevertheless, given its particular biological interest to human genetics, we do explore the population-level allele pattern content of the CEU and ASW *SLC24A5* skin pigmentation region in some more detail. From the broader genome-wide perspective we have to rely on less quantitative verbally expressed arguments. These draw intuitive connections between CE and various aspect of population genetic theory.

### Population discrimination

In order to infer population history, molecular geneticists conventionally look for specific genomic sites across individuals and search for changes in abundance or even fixation of an ancestral or derived allele [49]. Here we have taken a complementary strategy, exploiting various longitudinal patterns along an individual’s genome (i.e. the *rows* of Table 1 rather than just the columns). The assumption we make is that closely related individuals will be more likely to share these longitudinal patterns of genome composition (i.e. haplotypes), however complex they may be. One appeal of this approach is we do not lose the informational context provided by physically proximate SNPs.

In terms of the whole genome, we find CE allows all human populations to be discriminated, sometimes with little or no overlap. Using the Human HapMap3 dataset, the lowest CE is exhibited by the African American and other African populations and the highest CE by the Asian populations (Chinese and Japanese). The groupings resonate with published phylogeographic reconstructions based on F_ST_ and PCA analyses [50] but are computationally much quicker and cheaper, consuming only a fraction of CPU time. In broad terms, at heterozygosity levels less than a third, there is clearly a strong negative relationship between CE and heterozygosity. However, this observation does not explain the CE output, with populations of similar heterozygosity discriminated by differential CE. The population discrimination is robust across mammalian species (Figure 4). Runs of homozygosity are clearly an obvious compositional feature that will be exploited by *Gzip* to compress the SNP data string in the whole genome version of the CE analysis, but there are many others sources of regularity.

One appealing analytical implication of the genome-wide CE approach is that the different scales of the various informational regularities can be assessed simultaneously by a single metric, irrespective of their size, direction or crypticity. The genomes of domesticated species offer a useful model in which to explore the genomic consequences imparted by population histories characterized by bottlenecks and artificial selection, as the genetic similarity of the various breeds provides a relatively stable background against which various evolutionary forces can be inferred [51]. In each domestic species broadly similar patterns were observed. Genome-wide CE increased for populations likely to have been founded from a small number of founders, and it decreased for outbred populations expected to be highly heterogeneous. CE was plotted against heterozygosity showing that signals of population substructure were evident in non-human species (Figure 4).

### Genomic regions harbouring signatures of selection

Given the ability of whole genome CE to discriminate populations, we next explored within-genome structure to prioritise regions of particular biological interest. We used a high resolution sliding window expressed relative to heterozygosity and normalized by Z-score (CEhZ). The correction for heterozygosity means the CE differences are more likely to be attributable to the pattern in *order* of heterozygotes and homozygotes and less to changes in *proportion*. The approach integrates a combination of individual genome regularity in the context of population homogeneity in that region (Table 1). Regions of even quite complex composition will yield a compression peak by CEhZ if they are shared by many members of a given population. This feature discriminates the window-based CEhZ analysis (which compresses shared complex regions as well as shared simpler ones) from the whole genome CE (where compression will be most strongly influenced by low information content regions such as simple runs of homozygosity).

That is, for CEhZ not only would we expect to find peaks over regions characterized by shared runs of homozygosity (as exemplified by the *MSTN* locus in muscular Texel sheep which has recently been swept clean of genetic diversity), but other compositionally more complex regions as well. The application of a matrix structure that permits comparisons of the same genomic regions *across individuals* clearly connects the output to existing population-level metrics such as LD. However, CEhZ finds loci over many different kinds of compositional regularities in a manner that defies a simple summary. A more detailed examination of allele composition in the *SLC24A5* region in ASW, CEU and JPT reinforces the challenge of describing the mathematical nature of the compressible patterns exploited by *Gzip* (Figure 6). Irrespective of this, the data can be exploited to find commonalities and differences across any set of population groupings, in this particular case highlighting population substructure and showing the CEU and JPT are awarded similarly high CEhZ peaks for different reasons, and with different background HET.

In attempting to interpret the biological relevance of the compression peaks we examined the extent to which our new regions overlap with known signatures of selection. We find CEhZ successfully detects many of the known major signatures of selection in the various species. With regard to the human output we used [16] and found substantial overlap (Additional file 4: Data S2). The CE approach has the appeal of pinpointing particular genomic regions, such as coding sequence or parts of coding sequences, at very high resolution. In human populations the highlighted regions capture genes encoding proteins involved in skin pigmentation (*SLC24A5*), blue eye colour (*HERC2*), lactase persistence (*LCT*) and hair texture (*ECAR*).

A recent paper [18] detected a signature of positive selection in *SLC24A5* in CEU, GIH and TSI exactly concordant with our observations based on CEhZ. They used a new method (haploPS) which leverages 2 sources of information relating to haplotype length and structure. HaploPS is similar to EHH and XP-EHH except that it estimates the population frequency of the allele under question and identifies the haplotype sequence on which the selected allele sits.

Furthermore, the *LCT* gene encodes the lactase protein that allows milk digestion into adulthood. It is known to be under selection only in those European (CEU) and African (MKK) populations with an extensive pastoral history characterised by livestock domestication and an adaptation favouring regular milk and dairy consumption (Figure 7). The lactase signature recently detected in MKK was notable in that a combination of 3 computationally intensive measures had to be leveraged (fixation index, integrated haplotype score and cross population extended haplotype homozygosity) [52]. Moreover, in contrast to CEhZ which provides strong evidence for the exact gene, the alternative methods could resolve the region only to a relatively broad 1.7 Mb [52]. In our data the nature of the *LCT*-specific peak in CEU and MKK is visibly different, consistent with the purported evolutionary independence of the selection event [15].

New human population predictions unique to CEhZ imply hitherto unrecognised roles for a long non-coding RNA in European populations, a chromatin remodeller (*SCMH1*) in African populations and the *EDA2R* gene in Asian populations (Figure 8). The *EDA2R* is noteworthy in that it is present on the X chromosome which is not amenable to conventional analyses because it is hemizygous in males.

A number of CEhZ peaks are shared by all the human populations. We might speculate these represent genomic constraint at a much deeper taxonomic level, perhaps the branch point of modern humans from other primates. It is interesting to note that the more homogeneous Asian populations exhibit a frequency distribution of CEh Z-scores characterised by a low mean value, but a number of very extreme outliers compared to the more heterogeneous African populations (Additional file 2: Figure S2).

In the cattle divergent regions contrasting Angus (*Bos taurus*) and Brahman (*Bos indicus*) breeds aligned to previously described signatures of selection. The recently documented *PLAG1* is considered fundamental to tropical adaptation in Brahman cattle (Figure 9A, Table 2). New predictions for the cattle breed comparison include *EN1*, *EYA1* and *ARID4A* (Figure 9B, C, D). Overall, it appears CEhZ is a useful metric for exploiting intra-chromosomal heterogeneity in a rapid, straightforward fashion.

### Evolution and information compression

What are the biological implications of the CE values we have computed? As a first step, we defined the total parameter space (Additional file 5: Figure S3). This allows us to encapsulate the boundaries of the DNA informational universe. In turn, this universe enables us to envisage how real DNA sequence evolves at a whole genome level. We modelled ‘totally regular’ (highly compressible) by first sorting each individual SNP genotype prior to compression, and ‘totally irregular’ (uncompressible) through several different randomisation procedures set at both the individual and population levels that account for proportion and order. The initial randomisation provided by RAND1 breaks LD by scrambling the identical “0, 1, 2” proportions into chaotic order (Additional file 5: Figure S3).

This result shows that *in practice* LD serves to enforce data regularities (rather than irregularities) on real-world DNA sequences. The deeper randomisation provided by RAND2 (that borrows the proportions of 0’s and 2’s from the entire population, not the individual) is perhaps surprising. It suggests each individual genome possesses highly cryptic proportional regularities not present in the population at large (Additional file 5: Figure S3). We found that all populations in all species occupy a very specific zone, clearly converging at – or emerging from – a very well defined point in CE genomic space. They intersect at the point at which highly complex sequence most deeply explores the disordered space, without actually becoming chaotic. The overall uniformity of the shape of the output across all the populations/breeds of all the species, despite considerable compositional differences in both the density and functional bias of the SNP chip technologies, points to the very high robustness of the result. Nevertheless, SNP chips characterise highly variable (i.e. chaotic) regions, so the translation of this output to full genome sequence remains uncertain and should be a focus of future work. We were next interested in direction of travel through this space. Are we observing an emergence or a convergence from the point of minimum CE and maximum noise?

Our first attempt to answer this question was to examine the domestic species data. These species have a clearly identified progenitor which provides an unambiguous evolutionary sequence. However, SNP ascertainment bias confounds interpretation here. What we can say though, is that life - characterised by negative entropy [53] evolved from non-life which is usually characterised by high entropy. It is therefore tempting to assume that the more ancestral compositional states would have been more entropic. Further, another source of sequence data in a range of species supports this idea. In the context of particular protein coding sequences, we have previously noted that when genome-wide codon bias is quantified informationally, it is those proteins apparently most relevant to (or diagnostic of) the lineage under scrutiny that exhibit the lowest entropy.

Examples of these low-entropy ‘derived’ molecules include proteins influencing chloroplast physiology in plants, mitochondrial function in birds and hair formation in mammals [41]. Generalizing this broad line of reasoning (high entropy ancestral, low entropy derived) is appealing as it places representatives of the modern African populations as relatively basal (Figure 10), which seems to be consistent with the consensus “Out of Africa” hypothesis of modern human evolution [54]. In the future the hypothesis that derived genome sequences possess relatively low entropy could be validated using domestic species as a resource. One could compare the whole genome CE of the extant representative of the wild ancestor to various domestic populations. For example, we would predict the CE of the red jungle fowl genome, at an individual level, to be lower (i.e. more entropic) than individual genomes representing meat and egg producing domestic chickens. We would also predict population-level CEhZ sliding window scores to possess a more extreme distribution in the domestic breeds. Some of these CEhZ peaks would characterise signatures of selection for egg and meat production.

What else does this mean for our understanding of biological encoding systems? The phase transition between regularity and irregularity is theorised to be a high-impact zone of enormous computational power and evolutionary potential [55,56]. This interests us given a genome is a computing device made of nucleic acid that is the product of evolution. The overall position of all the human populations supports a controversial concept from complex systems science [55,57-61] that genomes are poised at or close to ‘The Edge of Chaos.’ This conclusion resonates closely with that of Kong et al., [62] who analysed 384 prokaryotic and 402 eukaryotic genomes using an novel regularity/order index called *ø* and based on averages of nucleotide distributions in a given sequence of pre-defined length.Figure 10 also summarise the possible mechanistic explanations for the various trajectories taken by the populations and individuals through information space, based on considerations of both the implications of our data modelling coupled with the real world mammalian genomes. We see different spatial impacts of LD and extent of outbreeding depending on the particular population under consideration.

### The meaning of CE in the context of population genetics theory

To finish, it is appropriate to more directly connect our CE work to existing population genetics theory, whose goal is to study the frequency and interaction of alleles and genes in populations. In population genetics theory, various evolutionary processes, particularly natural selection (in numerous guises), drift, mutation and gene flow are explored to make inference about population history. The Hardy-Weinberg principle says that the frequency of alleles will remain constant in the idealised absence of selection, mutation, migration and drift [63] and this provides a theoretical expectation (equilibrium) against which population level deviations from equilibrium (dis-equilibrium) can be quantified and subsequently interpreted. In Information Theory terms, the point of equilibrium corresponds to maximum entropy, and extent of dis-equilibrium reflects differing amounts of negentropy.

At a population level, nearby pairs of alleles have a high tendency to be correlated with each other (LD). In genetic ‘hitchhiking’ an allele at one locus rises to high frequency in a population because it is linked to an allele under selection at a nearby locus, not because it has been selected itself. The same phenomenon applies to genes under runaway sexual selection [64]. Clearly, this phenomenon culminates in population-level homogeneity (pattern) in allele combinations because of genomic similarity between individuals. Adding further dynamism, these *population*-level patterns are gradually broken by the *individual* cellular/molecular process of genetic recombination, but at a slow rate.

This ebb and flow of allele pattern formation and destruction among individuals can be exploited to detect the action of natural selection via selective sweeps, and to view the impact of migrations and founder effects. For example, it is well known that there is higher LD in Asian populations, presumably due to the founder effects that occur during migrations limiting the number of haplotypes. LD is often viewed by a decay plot e.g. [65], where it can be shown that deviation from equilibrium is considerably stronger for nearby loci. These decay plots are relatively extreme for Africans due to faster LD decay and correspondingly smaller haplotype blocks than in the comparison Asian and European populations. A number of existing metrics for selection (EHH, IHH) are based on considerations of local decay of haplotypes.

What does this mean for the various CE metrics and what are the phenomena that serve to underpin the patterns quantified by CE? Whole genome CE is computed on an isolated individual basis. The coordinates (i.e. shape and location) of the population cluster describes the data at the population level. However, given this is fundamentally an individual-level metric, its relationship to LD might not be straightforward. For example, other sources of (unknown) compositional regularity may apply including segmental duplications [66] and G4 motifs and structures [67]. It is also true *in theory*, that one can achieve the same compression efficiency for different reasons, but *in practice* we find that the accurate phylogeographic population-level clustering implies it is only similar related genome compositions that are awarded similar CE scores. Also, we know that the RAND1 modelling procedure serves to break LD and reduces CE (Additional file 5: Figure S3). Based on this reasoning, it is tempting to speculate that individuals with high CE presumably belong to populations that have even higher LD. This conclusion is clearly consistent with the population-level CE ranking we observe in Figures 1 ,2, 3 that mirrors known differences in LD between human populations, that is the Africans showing the least LD and the Asians the most LD, with the Europeans intermediate.

Next we will consider the relationship of this thinking to the particular genomic regions identified by the sliding window CEhZ. From our informational perspective, remnant population-level patterns can clearly be quantified by CEhZ, and contrasted across populations, no matter how cryptic or complex the genomic composition may be at an individual level. A detailed specific example is given by the CEU and GIH skin lightening signature of selection that resides over *SLC24A5* (Figure 6). At this stage, a confident determination of CEhZ’s exact biological origin – i.e. is there a particular compressible pattern diagnostic of natural selection versus genetic drift versus a founder effect? – is not possible.

That being said, there are clearly numerous reliable patterns present at very specific genomic regions in one population, but not another. Many of these have not been described before including those specifically overlying known functionally important parts of the genome, such as protein-coding genes, non-coding RNA and so on. These new discoveries may reflect the fact that the mathematical nature of the population-level patterns we have highlighted did not have to be specified *a priori*, unlike F_ST_ which is more tightly expressed. The guilt-by-association heuristic tells us there is some *bona fide* population-level meaning in those regions. It is our contention that a post-publication community effort and a range of techniques will be required to ascribe functional significance or not on a case-by-case basis. To expedite this process, we have uploaded the CEhZ tracks onto the UCSC genome web browser.

Slower LD decay in Asian populations seems consistent with our finding that the Asians possess extreme outlier peaks in CEhZ reflecting high homogeneity in certain regions not observed in the other populations (Additional file 2: Figure S2). It is worth pointing out that direct comparisons of two specified loci between populations are not apparent in decay plots, as all conceivable pairs are simultaneously plotted. By contrast, one strength of the CEhZ sliding window approach is we maintain the identity of the genomic region such that the population contrasts are directly comparable, and therefore biological interpretation can conveniently be made on a fine-grained regional basis. An example would be the European and Gujarati Indian selection events around the *SLC24A5* gene (Figure 6).

Finally, population genetic diversity has been quantified by allelic diversity – namely, the proportion of all copies of a gene made up of a particular variant [68]. The 1000 genomes consortium [69] showed that CEU, JPT and YRI possess many SNPs displaying substantial absolute differences in allele frequency, and that this ability to differentiate populations decays rapidly as one increases physical distance from genic SNPs. Our observations are consistent with some of these findings, namely the whole genome discrimination determined by CE, the concordance of some CEh peaks over genic regions in particular, and the elevation of African CE when examining coding SNPs only.

### Caveats

CE operates by exploiting regularities within a sequence regardless of the origin of the sequence. CE is not based on any theory of segregation inheritance, nor does it require knowledge of ancestry to phase the genotypes. In light of the strong correlation between F_ST_ and CE (r = 0.885) we conclude that CE accurately estimates genetic relatedness among populations without recourse to additional sources of information. The same conclusion is reached when CE is used a sliding window approach to capture genes under selection.

Because CE is a hypothesis-free pattern recognition method that detects regularities in segments of the genome, it is more in the spirit of the various haplotype-based methods, rather than single marker methods of population differentiation. The main weakness we have identified is - like EHH and F_ST_ - it does not allow for population stratification. Further work is required to formalise other strengths and weaknesses relative to existing methods.

Our implementation of CE requires the use of the gzip tool which incorporates the DEFLATE algorithm. This is included in all unix environments. However, other compression tools exist and could be used. Also, from the computational perspective, the sequential use of gzip (ie. one genotype sequence at a time) requires a great deal of parsing arguments and I/O operations. This is not an issue when compressing a whole matrix comprising genotype sequences from individuals within a population. Nevertheless, if we were to program the DEFLATE algorithm and perform CE analyses entirely in memory then the computation efficiency of CE analyses would be greatly improved.

## Conclusions

We have exploited the pattern recognition qualities of information compression efficiency to assess genome patterns within and between genomes. This approach recreates established phylogeography and highlights known signatures of selection. Some new regions with high population level scores overlying functional apparatus such as non-coding RNA have also been identified. These regions are not simple runs of homozygosity so direct assessments of this nature cannot identify them. Rather they are compositionally complex regions shared by individuals of a given population. We hypothesise some of these regions to be previously unidentified signatures of selection.

## Methods

### Exemplar data strings

To explore the patterns exploited by CE on an individual genome level we calculated the metric for several exemplar data strings of 30 SNPs. These ranged from simple and ordered to informationally complex and irregular. We chose two regular strings, each containing an even proportion of 0 s, 1 s and 2 s. The first contained a given SNP clustered together (i.e. 10 0 s followed by 10 1 s followed by 10 2 s), whereas the second had the 012 pattern repeated 10 times. These represent the two ways of generating the simplest patterns. We also generated a random sequence of 30 0 s, 1 s and 2 s. To model a population-level analysis we used 5 individual sequences per population.

### Datasets

We used seven SNP genotype datasets across five species (human, mouse, dog, sheep and cow). Genotypes were codified as “0” (homozygous wildtype or “AA”), “1” (heterozygous or “AB”), “2” (homozygous variant or “BB”), or “9” (missing data). These analyses were performed on publicly available data, each of which has previously documented ethics approval.

#### ***Human***

We obtained the HapMap-formatted genotype files corresponding to the HapMap phase III release 3 data (http://hapmap.ncbi.nlm.nih.gov/downloads/index.html.en). For the 11 populations, alleles from the ‘forward strand’ were used for the 22 autosomal and the X chromosome. This totaled 1,184 individuals and 1,457,897 SNPs. Details about number of samples genotypes for each population are given in: Table S1 of the original publication by the HapMap3 Consortium (http://hapmap.ncbi.nlm.nih.gov/downloads/presentations/nature09298-s1.pdf) [50].

We also utilized genotype data from the Human Genome Diversity Project (HGDP; http://hagsc.org/hgdp/files.html; [70-72]), comprising 660,918 SNPs and 1,043 individuals representing 51 different populations from 14 geographical regions of Africa, Europe, the Middle East, South and Central Asia, East Asia, Oceania and the Americas.

Additionally, we obtained the SNP genotypes from the Pan-Asia SNP Consortium (http://www4a.biotec.or.th/PASNP; [73]). The consortium provides the genotype data of 75 Pan-Asian and HapMap populations with 1,928 individuals, 54,794 SNPs on autosomal chromosome and 1,216 SNPs on chromosome X.

#### ***Mouse***

We accessed the data from Staubach et al., [74]. This comprises SNP calls from Affymetrix Mouse Diversity Genotyping Arrays. They were applied to 49 individual samples including 11 individuals from two populations each of *Mus musculus domesticus and Mus musculus musculus*. Additionally, there were single individuals representing 5 *Mus* outgroups.

#### ***Dog***

We used the SNP genotype data from Boyko et al., [75] comprising 60,968 SNP for 81 domestic animals, including a village dog, and four wild canids (wolf, red wolf, jackal and coyote).

#### ***Sheep***

We used genotype data from the International Sheep Genomics Consortium (http://www.sheephapmap.org/) described in [14]. From this resource, we used the genotype on 49,034 SNPs across 1,222 individual samples from 9 sheep populations including: South-East Europe (n = 177), North-East Europe (n = 209), Middle East (n = 196), Africa (n = 135), Americas (n = 126), Asia (n = 210) and feral sheep (n = 52).

#### ***Bovine***

We used genotype data from the Cooperative Research Centre for Beef Genetic Technologies (http://www.beefcrc.com/) originally described by [23]. From this resource, we used the genotype on 729,068 SNPs across 1,800 individual cattle samples comprised of 200 individuals from each of the following 9 breeds: Angus, Brahman, Belmont Red, Charolais, Drought Master, Hereford, Santa Gertrudis, Shorthorn and Tropical Composite.

### Compression efficiency

For each of the individual samples described above, we built a single-column file with the genotype profile across all the available SNPs sorted by chromosome (first the autosomal chromosomes and then the X chromosome) and by genome location within chromosome. Kolmogorov complexity can be approximated through the use of real world compression algorithms [9]. To apply the concept here the size of the genome SNP file (s) in bytes were noted before and after compression using the *gzip* application tool of UNIX systems http://www.gzip.org/.

The application *gzip* is based on DEFLATE, a lossless data compression algorithm originally described by [76]. DEFLATE exploits two compression strategies, the LZ77 algorithm and Huffman coding. The LZ77 algorithm is a sliding window approach that identifies exact repeats and encodes their presence with two numbers, a distance and length. Huffman coding builds a binary tree representing the overall proportions of each element in the data stream, with the most frequent characters being denoted by short path lengths. *Gzip* has previously been used to cluster data on compression e.g. [10].

Compression Efficiency (CE) was computed as follows: $\mathrm{CE}=\frac{S_{B}-S_{A}}{S_{B}}$ where *S*_*B*_ and *S*_*A*_ represent the size of the SNP genotype file in bytes before and after compression, respectively.

### Relationship between CE and F_ST_

To formalise the concordance between F_ST_ and CE measures of population differentiation, we computed the correlation between the two metrics as applied to the human HapMap data [50]. First, we acquired the F_ST_ values for the 55 pair-wise comparisons from the 11 populations using Table S6 [50]. We then computed the absolute difference in population-level CE for each pair and correlated this value against F_ST_.

### Coding *versus* Non-coding analysis

For the SNPs in the Human HapMap dataset, we used the UCSC snp131 annotations [77] which has, as part of its annotation set, the genomic location of each SNP. Using these annotations, we parsed the list into six mutually exclusive groups: Group 1 – annotated with missense; nonsense; frameshift; splice-3; Group 2 – coding-synonymous; Group 3 – untranslated 3’ end; untranslated 5’ end; Group 4 – near-gene 3’ end; near-gene 5’ end; Group 5 – intron; Group 6– unknown (i.e. not in a protein coding gene). The parsing was done in the order above – i.e. annotations from Group 1 received precedence, followed by Group 2 through to Group 6. The “unknown” group only contained unknown SNPs; but Group 1 contained those annotations plus others if the SNPs happens to overlap multiple features. A general comparison between coding and non-coding was performed by combining Groups 1 to 3 (coding) and Groups 4 to 6 (non-coding). Intersections were performed using the UCSC backend tool bedIntersect, with the hg18 reference genome and canonical Refgene annotations.

### High resolution window-based search for genomic regions under constraint and signatures of selection

Using the human HapMap3 data and the Angus and Brahman bovine data, we surveyed the entire genome using sliding overlapping windows of 50 consecutive SNPs. Hence, each window shared 49 SNP with its neighboring windows. These windows corresponded to approximately 0.1 Mb and 0.2 Mb for the human and bovine data sets, respectively. Because CE was highly influenced by the window-wide percent heterozygosity (%*Het*), CE was adjusted for %*Het*, termed CEh and computed as follows:

$$\text{CEh}=\frac{\mathrm{CE}}{\text{\%Het}}$$

This heterozygosity correction partly accounts for regularities attributable to allele proportion, leaving those regularities attributable to allele order or pattern. Plotting CE against HET, as opposed to Shannon’s Entropy or, say, Principal Component 1 is appealing from a biological perspective. After all, HET is a basic statistic whose relevance to genetics is well recognized.

For the window-based search, we applied CEh to the data matrix comprising the genotypes of the 50 SNPs in the window (in columns) across all the samples in that population (in rows). We elected to use 50 SNP windows because this yields a convenient size when translated into genomic bases. The most sensitive window size will vary with the particular genomic region under investigation and the particular population, and would need to be optimized on a case by case basis.

The incorporation of population-level data means that even genomic regions that are complex (relatively uncompressible) in isolation will still be detectable by a compression peak because they are shared by many members of a given population. This contrasts with a sliding a window performed on an isolated individual basis, which would exclusively prioritize low information content areas (e.g. highly compressible runs of homozygosity - string of 0 s or 2 s). The method and its implications are summarized in Table 1.

In order to allow for unbiased comparisons within and across populations, the CEh was normalized. We computed its z-score by subtracting the genome-wide average CEh and dividing by the genome-wide standard deviation. The X chromosome was normalized separately due to its homozygosity.

To obtain high resolution windows of CEh each SNP was assigned the average z-score CEh (CEhZ) of all the windows in which it was represented – i.e. each SNP was assigned a value that was derived from the average of all CE sliding windows that it overlapped (which for all but those SNPs at chromosome termini was 50). To pinpoint regions of extreme allele order (i.e. high CEhZ scores), SNPs with CEhZ scores 3fold higher than the average CEhZ score across each chromosome were identified. SNPs within 20 Kb of each other were then clustered and BED files of the regions of interest generated for the immediate incorporation into the genome browser. To expedite this process, we have uploaded the CEhZ tracks onto the UCSC genome web browser (http://www.genome.ucsc.edu/cgi-bin/hgGateway).

### Modeling genomic information content

In order to ask further questions about the evolution of genomic information content we undertook an *in silico* modeling approach. We wanted to better understand the biological implications of the observed genome-wide CE scores. CE lends itself to modeling order and disorder in a convenient manner that incorporates any regularities present, irrespective of whether they derive from proportional bias or order, or indeed the size, direction or crypticity of the various longitudinal patterns.

A combination of randomization (to generate chaos) and ranking (to generate order) allowed us to define the outermost boundaries of the parameter space in which to embed and visualize the real-world data. For each of the genotypes built for each individual in the human HapMap3 data, we explored two randomization schemes, each of which serves to systematically destroy two different sources of regularities.

In the first scheme (RAND1), we generated a random sequence that preserved the exact same genotype proportions as the real individual-level data. In the second scheme (RAND2), we explored a deeper level of randomness. RAND2 sequences were generated from a random sequence that preserved the exact same proportion of HET observed in the real sequence of the individual under scrutiny, but the proportion of the two homozygous were obtained from those observed in the entire population where the individual came from. For instance, if the sequence of the individual in question was heterozygote for 20% of the SNPs, and the entire population showed a ratio of 3 to 1 between the homozygotes of the first allele (‘A’) and the second allele (‘B’), then the RAND2 sequence for this individual will comprise 60% ‘AA’, 20% ‘AB’ and 20% ‘BB’.

Finally, for each individual, ten RAND1 and ten RAND2 sequences were generated and the average CE recorded to be compared with the CE of the real sequence. The application of this procedure means that for every individual point on the plot, there is a corresponding RAND1 and RAND2 value. These can be used to formalize observed versus expected CE scores for the various individuals and populations.

## Competing interests

The authors declare that they have no competing interests.

## Authors’ contributions

AR and NJH conceived of the study. NJH drafted the manuscript. AR, RJT, LRPN and SM performed the analyses. RJT, NJH, JK, LRPN and AR biologically interpreted the analyses. AR coordinated the project. All authors read and approved the final manuscript.

## Supplementary Material

Additional file 1: Figure S1 Compression Efficiency in Coding and Non-Coding Regions. The impact of SNPs in coding regions: For the Human HapMap population, relationship between (A) heterozygosity and (B) compression efficiency using either all 1.4 M SNPs (x-axis) or using only the 56,571 SNPs located in coding regions (y-axis). The straight line represents the line of unity. As extensively reported, there exits less heterozygosity in coding regions with the magnitude of the decrease in heterozygosity similar in all populations. A decrease in heterozygosity coupled with an increase in compression efficiency is only observed for the African populations. For the other populations, the compression efficiency using only coding SNPs is unchanged or slightly smaller. Click here for file

Additional file 2: Figure S2 A Genome-wide frequency distribution of CEh peaks in human populations. The highly heterogeneous ‘ancestral’ African populations have an even distribution of heterozygosity corrected compression efficiency peaks possessing a relatively high mean value. In contrast, the ‘derived’ Asian populations with a smaller effective population size have a distribution characterised by a number of very extreme heterozygosity corrected compression efficiency peaks against a substantially lower background. B The distribution of heterozygosities of the heterozygosity corrected compression efficiency peaks clearly shows many peaks have substantial heterozygosity and therefore cannot be attributed to simple runs of homozygosity. In these box and whisker plots, the mean average is the solid black line within the box, the boundaries of the box are the 25% and 75% quartiles, the whiskers are 95% confidence intervals and the solid black dots represent outliers. Click here for file

Additional file 3: Data S1 The genomic location of the CEhZ peaks in the various human populations. Click here for file

Additional file 4: Data S2 Those CEhZ peaks in common with regions identified by Grossman et al [16]. Click here for file

Additional file 5: Figure S3 Positional Orientation of Real Genomes in Information Space. The randomization strategy termed “RAND2” can be explored beyond the limits of heterozygosity observed in the HapMap population (i.e. 23.9% to 31.2%) across its entire parameter space i.e. 0 to 100% (blue line). Zooming into the real genomic data we discovered that all sequences including the modelled RAND1 (represented in red) converge towards a single point corresponding to that where a minimum compression efficiency (maximum noise) can be obtained. From a complex systems science perspective this point in informational space is a graphical representation of ‘The Edge of Chaos.’ This phase transition is important because computational capacity and evolutionary potential are thought to be maximised here. Click here for file
