# Supplementary figures and images for: Information compression exploits patterns of genome composition to discriminate populations and highlight regions of evolutionary interest
Source: BMC Bioinformatics. 2014 Mar 7;15:66. doi: 10.1186/1471-2105-15-66 (PMC4015654; doi:10.1186/1471-2105-15-66)

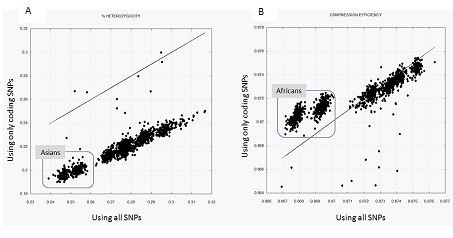

Supplement: Additional file 1: Figure S1 — Compression Efficiency in Coding and Non-Coding Regions. The impact of SNPs in coding regions: For the Human HapMap population, relationship between (A) heterozygosity and (B) compression efficiency using either all 1.4 M SNPs (x-axis) or using only the 56,571 SNPs located in coding regions (y-axis). The straight line represents the line of unity. As extensively reported, there exits less heterozygosity in coding regions with the magnitude of the decrease in heterozygosity similar in all populations. A decrease in heterozygosity coupled with an increase in compression efficiency is only observed for the African populations. For the other populations, the compression efficiency using only coding SNPs is unchanged or slightly smaller. [file 1471-2105-15-66-S1.tiff]

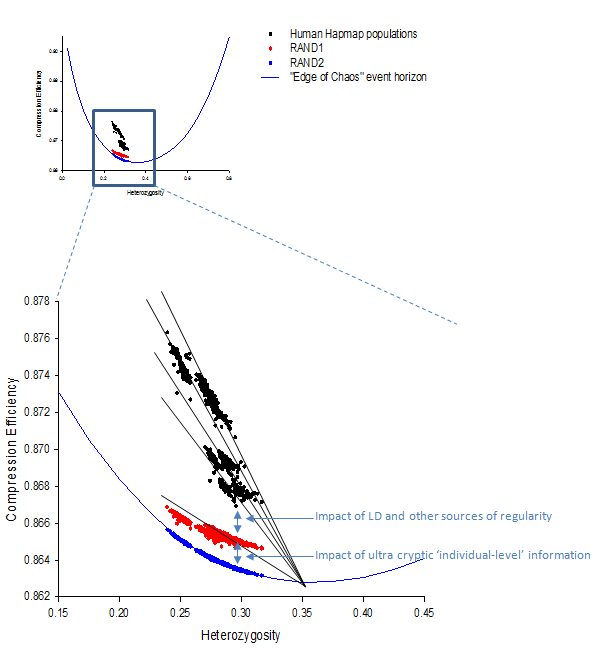

Supplement: Additional file 3: Data S1 — The genomic location of the CEhZ peaks in the various human populations. [file 1471-2105-15-66-S3.tiff]
